# Measuring psychosocial outcomes: is the consumer or the professional the best judge?

**DOI:** 10.1111/ecc.12048

**Published:** 2013-02-25

**Authors:** C Paul, R Sanson-Fisher, M Carey

**Affiliations:** The Priority Research Centre for Health Behaviour, Faculty of Health, University of NewcastleCallaghan, NSW; Hunter Medical Research InstituteNewcastle, NSW, Australia

**Keywords:** cancer, psychosocial care, quality of care, consumer, physician

## Abstract

In this review, we explore *professionally-driven* and *consumer-driven* paradigms in measuring psychosocial outcomes for cancer care. Early measures of psychosocial well-being focussed on clinically-derived concepts of dysfunction. Recent literature reflects a paradigm shift toward a *consumer-driven* approach to the conceptualisation and measurement of psychosocial well-being. The key distinction between the two approaches rests on whether the professional or consumer retains judgement authority and raises the question of whether it is necessary to include both perspectives in research and practice. Research is proposed to clarify our interpretation of these approaches with a view to devising novel interventions to benefit patient well-being.

## Introduction

### The importance of measuring psychosocial well-being

Over the past two decades as cancer survival rates have improved (Australian Bureau of Statistics 2004) there has been increasing interest in finding mechanisms to improve patients' and survivors' psychosocial well-being (Ross *et al*. 2002). Psychosocial well-being is a holistic term which encompasses psychological, physical, social and spiritual health (National Comprehensive Cancer Network 1999; Rowland *et al*. 2006).

There has been increasing attention to the development and testing of measures of psychosocial well-being from the development of measures of depression during the 1960s (Beck *et al*. 1961; Zung 1965) to the development of unmet needs scales for cancer patients in recent decades (Pearce *et al*. 2008). Psychosocial measures cover a range of concerns from specific types of psychological disturbance such as anxiety as measured in the Hospital Anxiety and Depression Scale (HADS) (Bjelland *et al*. 2002) and the State-Trait Anxiety Inventory (STAI) (Endler & Kocovski 2001), through to depression such as the HADS, the Depression Anxiety Stress Scales (DASS) (Page *et al*. 2007) and the Centre for Epidemiologic Studies Depression Scale (CES-D) (Radloff 1977). Psychosocial measures also include broader conceptualisations of general health in the Short-Form Health Survey (SF-36) (McHorney *et al*. 1993); psychological well-being in the General Health Questionnaire (GHQ) (Goldberg 1979); quality of life in the European Quality of Life (EuroQol) (Brazier *et al*. 1993); and also unmet needs as measured in the Supportive Care Needs Survey (SCNS) (Bonevski *et al*. 2000) and the Cancer Survivors' Unmet Needs (CaSUN) (Hodgkinson *et al*. 2007).

Such increased attention to the measurement of psychosocial health is considered to reflect a key change in social concern or core societal values (McDowell 2006). Indeed, psychosocial well-being is often considered an integral part of good health (Anderson 1992; Sobel 1995) and there is an emerging body of literature on interventions to improve psychosocial care for cancer patients (Meyer & Mark 1995; Fawzy 1999; Ross *et al*. 2002; Rehse & Pukrop 2003; Boesen *et al*. 2011). These efforts to improve psychosocial well-being for cancer patients require robust and effective measures in order to establish prevalence of psychosocial concerns, and to evaluate the effectiveness of interventions.

### Two valuable perspectives: *consumer-driven* and *professionally-driven* approaches

Various ways of comparing and classifying measures of psychosocial well-being have been proposed, including purpose, scope and methodological approach (McDowell 2006). The scope of many of the earliest measures of well-being focussed on clinically-derived concepts of dysfunction, such as depression (Larson *et al*. 2010). More recent views about patient well-being have broadened to encompass a range of physical, social and spiritual aspects of well-being such as those captured in quality of life (QoL) scales [e.g. Functional Assessment of Cancer Therapy (FACT); Cella *et al*. 1993]. These QoL scales sometimes incorporate a consumer perspective in conceptualisation and development. The relatively recent emergence of measures of unmet need reflect a further paradigm shift in the conceptualisation and methodology for developing measures of psychosocial well-being. Measures of unmet need place a much greater emphasis on the consumer perspective rather than the professional perspective (Sanson-Fisher *et al*. 2009). Emphasis on the role of healthcare professionals in the conceptualisation, development and application of these measures has been described as a ‘top-down’ approach, while a consumer emphasis has been termed a ‘bottom-up’ measurement approach (McLachlan *et al*. 2001; Sanson-Fisher *et al*. 2009). A number of studies of psychosocial care have used both approaches to measurement (Macvean *et al*. 2007) suggesting that the two approaches may be considered as either complementary estimates of a single construct or as measures of quite separate or different constructs. Many other studies of psychological well-being retain a *professionally-driven* approach to measurement (Alexander *et al*. 2010).

## Method

### Why is it important to distinguish between the two approaches?

A clear understanding of the difference between a *professionally-driven* and a *consumer-driven* approach is crucial to our choice of tools for psychosocial measurement, whether assessing research outcomes or to triaging patients into supportive care. There is a need for clarity regarding the circumstances where an accurate and complete assessment of psychosocial well-being requires both perspectives.

To date, very little of the psychosocial literature has been devoted to clarifying this issue. While both the *professionally-driven* and *consumer-driven* approaches have merit and value, it is argued here that the two approaches operate within differing paradigms. This paper will describe the underpinnings of the two approaches and the implications of each approach for both research and clinical practice. Suggestions for research will be provided, which may help in understanding how these approaches can be used to provide the greatest possible benefit to patients and aid clear interpretation of psychosocial measurement outcomes.

## Results & Discussion

### What characterises a *professionally-driven* versus a *consumer-driven* approach?

The key distinction between the two approaches rests on whether the health professions or the consumer retain the greater degree of authority and control in judging well-being. While the measurement of psychosocial health generally involves a subjective judgement (the view of a clinician, patient or family member), *professionally-driven* approaches give greater relative weight to the view of the health professional. A *professionally-driven* approach assigns the health professional the role of defining the nature and extent of wellness or disease. A *consumer-driven* approach allows the consumer to define what constitutes wellness. A *consumer-driven* approach emphasises patient decision making about need for care or intervention, while a *professionally-driven* approach seeks to classify individuals or populations on the assumption that ‘caseness’ defines whether or not intervention will be of benefit.

**Figure 1:**
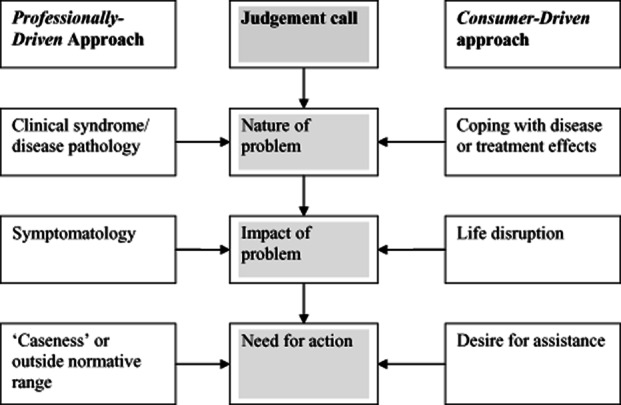
Contrasting the *professionally-driven* and *consumer-driven* approaches to judgements about psychosocial well-being.

The two approaches give rise to fundamental differences in the major ‘judgement calls’ involved in decision making about psychosocial care (see Fig. 1). These judgements relate to the: nature of the problem, the impact of the problem and the need for action to ameliorate the problem. The two approaches will be contrasted here, using selected examples from scales designed for or frequently used in studies of cancer patients or survivors.

### Contrasting the *professionally-driven* approach with the *consumer-driven* approach

#### The nature of the problem

A *professionally-driven* approach is largely grounded in the medical model of disease where a trained expert (e.g. psychologist) is required to identify or diagnose a problem or condition which has a specific aetiology and pathology (Shan & Mountain 2007). The biomedical model underlying a *professionally-driven* approach operates on the premise that disease is ‘accounted for by deviations from the norm of measurable biological variables’ (Engel 1977). This approach assumes that there is sufficient stability in the pattern of symptoms to diagnose an underlying pathology or clinical syndrome. Within such a paradigm, the measurement of psychosocial distress involves deciding whether or not the individual meets independent criteria (e.g. DSM IV) or falls outside normative population data. A *professionally-driven* approach to psychosocial well-being is, therefore, generally defined in terms that readily relate to the training of existing health professions, thereby assigning expert status and control to those professions.

Examples of a *professionally-driven* approach to assessing psychosocial well-being include an interview with a clinical psychologist or an assessment by an allied health professional. An outworking of this approach is reflected in scales which mimic clinical judgements about psychosocial well-being [e.g. Beck Depression Inventory (BDI), STAI]. For these scales, professional opinion is the basis for generating items and scores are validated against clinical judgement or standards. For example, the CES-D (Radloff 1977) was developed to quantify the main symptoms of depression identified in the psychiatric literature (McDowell 2006). Similarly, the conceptualisation of the EuroQoL instrument was based primarily on the views of clinicians and behavioural scientists (Aaronson 1988; Aaronson *et al*. 1991; McDowell 2006).

A *consumer-driven* framework defines psychosocial health from the patient's perspective as a negative effect arising from the disease or the treatment. Phenomenological analysis reveals that patients experience illness as a disruption of the lived body rather than dysfunction of a biological body (Tombs 1993). This suggests the biomedical model is incomplete as a basis for understanding psychosocial well-being, and the subjective patient perspective can provide relevant and valid information in its own right. In a *consumer-driven* approach, the cancer survivor is accorded ‘expert’ status during the conceptualisation and development of the measure, using their experiences to generate items. Unwellness is identified via comparison with the individual's own experiences or preferences, rather than comparison with independent criteria or the norms of a broader population. Measures of unmet need such as SCNS (Bonevski *et al*. 2000), SUNS (Campbell *et al*. 2011) & CaSUN (Hodgkinson *et al*. 2007) are designed to identify specific issues which patients see as requiring some action or resource in order to attain optimal well-being (Sanson-Fisher *et al*. 2000). The *consumer-derived* nature of these measures is evident in domains or factors such as information needs, financial needs and relationship needs which are not evident in the *professionally-driven* measures. Thus, a *consumer-driven* measure such as the SUNS is more likely to identify a desire for assistance that is relevant to the patient, regardless of whether there is a health professional trained to deal with the need.

It should be acknowledged that some scales have been developed using both a *professionally-driven* and a *consumer-driven* perspective. The FACT QoL scale (Cella 1992), is potentially an example of a hybrid approach in that the patient's subjective judgement about the degree of impact the disease has had is considered to be fundamental to the concept and to scale development (Cella 1992). The FACT does, however, take a *professionally-driven* approach in terms of classification of an individual's with reference to population norms. Such a truly hybrid approach does appear, however, to be relatively rare in existing psychosocial measurement for cancer patients.

#### The impact of the problem

The biomedical model assumes that disease is an ‘undesirable deviation or discontinuity’ that ‘gives rise to the need for corrective actions’ (Engel 1977). This facilitates standardisation of diagnoses and therefore, choice of appropriate treatment regimes (Widiger & Samuel 2005). A *professionally-driven* approach to psychosocial measurement will as a result, focus on describing and quantifying recognised symptoms. For example, QoL measures such as the Functional Living Index for Cancer (FLIC) quantify the degree or frequency of a symptom without reference to whether that experience is of importance or value to the patient:

How much of your usual household tasks were you able to complete? (FLIC) (Schipper *et al*. 1984)

In contrast the *consumer-driven* approach seeks the patient's views about what type and degree of impact constitutes a problem, and importantly, assumes the patient is accurate. The patient is considered to have the right and ability to determine what constitutes a problem regardless of their level of symptomatology. For example, a motor impairment in exchange for a decrease in pain might be acceptable to one patient, but unacceptable to another who requires fine motor skills in his or her work. Another patient may consider symptoms of anxiety to be reasonable given their diagnosis, with no associated desire for treatment or assistance. A *consumer-driven* perspective allows patient values to be taken into account in assessing what is an acceptable symptom, restriction or inconvenience. In such a paradigm, a specific issue (e.g. need for help coping with feelings of grief) is placed under the spotlight rather than the person being identified as problematic or dysfunctional.

#### Need for action

*Professionally-driven* approaches to measurement are designed to classify patients as being cases (e.g. clinically depressed or anxious), non-cases or borderline (Bjelland *et al*. 2002; Ng *et al*. 2007). In developing scales, judgements about reliability and validity are determined by the specificity and sensitivity of the scale judgement compared with a gold standard. The gold standard is often a psychiatrist's determination of whether or not the individual should be classified as a case (Steer *et al*. 1999), for example, as measured in the HADS (Bjelland *et al*. 2002), the Depression Anxiety and Stress Scale (Nieuwenhuijsen *et al*. 2003; Mitchell *et al*. 2008), the Brief Symptom Inventory (Zabora *et al*. 2001; Meachen *et al*. 2008) and the Beck Depression Inventory (Furlanetto *et al*. 2005). In the case of scales measuring physical functioning and general health, the validity of the scale may be assessed in terms of whether it discriminated between patients with different types of medically diagnosed conditions (SF 36) (McHorney *et al*. 1993). Therefore, the development of a *professionally-driven* measurement approach places judgement into the hands of professionals with little or no reference to consumer or patient perspectives on what constitutes health. In a practice setting *professionally-driven* data are suited to the triaging of individuals to particular types of care which already exist as professional disciplines.

A *consumer-driven* framework (Ruland *et al*. 2005) considers principles of independence, self-determination and control to be fundamental to positive outcomes (Maclean 1995). The patient chooses whether or not they desire assistance and to what degree care or support is of personal importance (e.g. SCNS, SUNS). Patient values are perceived to be central to successful behaviour change as per a motivational interviewing framework (Miller & Rollnick 1991). Studies of the therapeutic alliance model, suggest that psychosocial outcomes are enhanced when the patient and provider pursue a collaborative approach to care delivery (Carroll *et al*. 1996; Castonguay *et al*. 1996). This approach owes much to the growth in the consumer movement and the acknowledgement that patients have the right to be involved in their care and decision making. In the example of psychiatric treatment, proponents of consumer empowerment compare the sometimes debilitative consequences of psychiatric treatment (dependence, self-doubt and loss of control) with the recovery-promoting effects of consumer-led mutual support approaches (Maclean 1995). Therefore, a *consumer-driven* concept of psychosocial well-being does not cede control to the professional. Within this paradigm the frequency with which the problem occurs in the population does not determine need for help. Rather, the individual constitutes his or her own reference point. The result of a *consumer-driven* approach is also likely to be the identification of a number of needs which relate to deficits in the behaviour or responsiveness of the healthcare provider or the actions of the system. The patient is then in the position of raising an awareness of how the system must change to provide better psychosocial outcomes for patients.

### Reconciling a *professionally-driven* approach with a *consumer-driven* approach

A consideration of the differences between the *professionally-driven* and *consumer-driven* paradigms raises the question of whether it is necessary to include both perspectives in research and practice, and the degree to which these outcomes are complementary. Since the advent of *consumer-driven* psychosocial measures in the 1980s (Sanson-Fisher *et al*. 2009), *consumer-driven* measures have rarely been used as the sole outcome measure in intervention trials. A brief search of the unmet needs literature from 2004 to 2010 suggests that a combined approach whereby the *consumer-driven* measure (unmet needs) is used alongside a *professionally-driven* measure on most occasions (Millar *et al*. 2010). However, many studies of psychosocial well-being in cancer patients from the same period include only *professionally-driven* measures (Larson *et al*. 2010). The combination approach might be considered the best of both worlds, in that both the professional and consumer perspectives are taken into account. However, there is potential for redundancy, complementarity and conflict when the two measures both address a common issue, such as depression. This can occur for example, when an individual's score on depression subscale of HADS is compared with their stated need for help on SUNS items such as ‘dealing with feeling depressed’, or ‘coping with feelings of despair’.

The implications and relative merits of the two approaches can be assisted by examining hypothetical scenarios in which both measurement approaches are used to assess a patient in relation to feelings of depression. The cells in Table 1 represent the potential findings for patients a, b, c and d using both approaches. This illustration indicates that the two approaches might agree or disagree as to the need for psychosocial support. As described below, there is a need for careful consideration of how one might proceed, particularly when the two approaches provide potentially discordant information. For both the researcher and a clinician, this can present a problem.

**Table 1:** A representation of potentially concordant and discordant findings for *professionally-driven* versus *consumer-driven* measurement regarding feelings of depression

*Consumer-driven* judgement (e.g. SUNS)	*Professionally-driven* judgement (e.g. HADS score)	

	Depressed	Not depressed
Desire for help with feelings of depression	*a*	*b*
No desire for help with feelings of depression	*c*	*d*

***Group a***: This group is comprised of people who score as ‘cases’ on a depression measure such as HADS *and* also indicate a high need for help (e.g. SUNS emotional needs ‘dealing with feeling depressed’, ‘coping with feelings of despair’). A person who falls within this group may be aware of symptoms such as intense tiredness and sadness, and feel that assistance might help with managing their symptoms.

***Group b***: These people fail to meet the case threshold on the *professionally-driven* measure of depression (e.g. HADS), yet identify a need for help with feelings of depression on unmet needs scales such as the SCNS. Despite not reporting significant symptoms on a *professionally-driven* scale, such an individual may express a need for help due to lack of personal or social resources for dealing with feelings which are perceived to be challenging.

***Group c***: This group includes people who may be classified as cases of depression on a *professionally-driven* scale such as HADS, yet report no desire for help on a measure of unmet need. For example, a patient may report poor sleep and an ongoing lack of enjoyment of life, yet consider this situation to be ‘normal’ for them due to having prior untreated depression. Alternatively, a group c patient may prefer to rely on his or her internal coping mechanisms despite experiencing burdensome symptoms.

***Group d***: A non-case (i.e. low score on HADS) who does not indicate a desire for help on any emotion-related items of unmet need would fall into group d: This group has low depressive symptomatology and no reported desire for help.

#### Interpreting accord & discord when both approaches are used

##### Accord – groups a & d

For the above scenario, there is concordance between *professionally-driven* and *consumer-driven* measurement for patients in groups a and d. In the case of group a, both measurement approaches suggest a need to offer available evidence-based therapies for depression. The two approaches could be considered complementary, as the range of needs assessed on the SUNS may identify factors contributing to the depression and so assist with triage into relevant care. For group d both approaches suggest no cause for concern or action and one of the measures is potentially redundant either as a trial outcome or triage tool.

##### Discord – groups b & c

In the case of group b patients (those who want help but are not ‘cases’), the two approaches are essentially in conflict. For this group, using a solely *professionally-driven* approach may result in the patient's desire for assistance going unnoticed. Here, a *consumer-driven* approach is more likely to result in linking the patient to a range of support options. If we assume that there is available effective treatment to improve psychosocial well-being, group b (want help but are not ‘cases’) may experience preventable distress. This group may require additional support from relatives or frequently attend the General Practitioner (GP) if their perceived need for assistance with feelings of depression is not identified or not addressed. For group c, insistence on diagnosis and referral could improve depressive symptoms, which if untreated might have resulted in a burden on the patient, family and health system. On this basis, it would seem potentially useful to employ both measurement approaches and extend treatment to all who may benefit either on the basis of ‘caseness’ or perceived need.

Group c patients do not want help but may potentially derive benefit if they were to receive such help. The *professionally-driven* approach might result in the healthcare provider exploring the problem, strongly recommending therapeutic options. Psychosocial intervention trials suggest it is difficult to achieve an improvement in psychosocial outcomes (Brown *et al*. 2006). There is also very limited evidence of predictive validity for most *professionally-driven* and *consumer-driven* measures, for example, in terms of increased healthcare utilisation (Keeley *et al*. 2008). That is, few studies have provided sound evidence regarding whether there is a link between psychosocial variables such as depression and outcome data such as 5-year survival rates or disability-adjusted life years (DALYs) (Scherer & Herrmann-Lingen 2009). For group c, an arbitrary medicalisation of a transitory response to their experience may be of no benefit to psychosocial health or potentially interfere with natural coping mechanisms. Also, if the patient does not perceive a problem, they may not accept the referral or may drop out of therapy early, reducing the likelihood of realising potential benefits.

#### Research which may lead us forward

While it is unlikely that there are simple answers to the dilemma of how best to use *consumer-driven* and *professionally-driven* approaches to measuring psychosocial well-being, some research questions emerge which may help to progress the field:

It would be helpful to identify whether those who want help but are not ‘cases’ can receive benefit from traditional psychosocial interventions. Currently most trials have restricted intervention to those who meet criteria for ‘caseness’ (Sharpe *et al*. 2004). Clarification of the role of *consumer-driven* approaches to measurement would be provided by studies with sufficient power to compare the relative long-term benefit of psychosocial interventions for both cases and non-cases who report a desire for assistance.

Further exploration of such questions may help to provide some direction about the ambivalent results of psychosocial intervention trials. Many of the published studies using unmet needs as an outcome measure have not produced a change in these outcomes (Kato *et al*. 2008; Eriksson *et al*. 2010). It may be that *consumer-driven* outcomes such as unmet need are not responsive to any psychosocial intervention; rather they follow a natural history for each individual, regardless of the provision of support.

Studies which focus on those who would be considered cases under a *professionally-driven* approach but do not want help would also help to clarify the relative value of incorporating a *consumer-driven* measurement approaches with the more traditional *professionally-driven* measures. It is important to identify whether such people will suffer preventable burden if they are not strongly encouraged to receive help. A study which assesses the acceptability and impact of allocating such people to either an intensive attempt to identify acceptable support options versus a monitoring-only approach would identify the degree to which an expression that help is not needed can be taken at face value. More efficient and effective approaches to care delivery would result from robust answers to such a research question.

An exploration of the dimensions and content of *consumer-driven* approaches as a tool for intervention development may also be of value for advancing research into psychosocial well-being. Relatively few (if any) interventions have been designed to address the wide range of issues contained in these measures. We are yet to see whether interventions specifically designed from a *consumer-driven* approach are feasible and potentially more effective (for some patients) than current approaches to intervention development.

It is also likely to be beneficial to conduct review and secondary data analyses to identify the relative proportions of concordant versus discordant responses in studies which have used both approaches to measurement. If concordance is high (i.e. groups a & d constitute a high proportion of participants) this suggests the two approaches may be measuring the same construct. If this is the case, only very selective use of *consumer-driven* measures is required where identification of specific unmet needs may help with triage to care or interpretation of study results. If it is the case that discordance is high (groups b & c), it is likely that a *consumer-driven* approach may be measuring something quite different to that of *professionally-driven* approaches, and both types of measures are therefore necessary to comprehensively measure psychosocial well-being.

## Conclusion

The *consumer-driven* approach to measuring psychosocial well-being has the potential to provide valid and useful data about patients' need for and responses to psychosocial care. Considered exploration regarding the impact of incorporating these approaches alongside more traditional *professionally-driven* measurement approaches has the potential to increase the effectiveness and efficiency of psychosocial interventions. The current lack of clarity about how to interpret discordant data emanating from combined use of these two approaches, poses a set of dilemmas.

The proposed research questions, if addressed in a robust manner, can identify whether there is any benefit in considering these approaches in a complementary manner, and open up potentially novel interventions which may benefit patient well-being.
